# On the Various Means of Preparing and Exhibiting the Croton Tiglium, Used by the Natives of India

**Published:** 1823-05

**Authors:** W. Evans

**Affiliations:** Surgeon R.N.


ART. IX.—*On the various Means of Preparing and Exhibiting the Croton Tiglium, used by the Natives of India.* By W. EVANS, Esq. Surgeon R.N. Communicated by R. BAMPFIELD, Esq.

THESE nuts have been used medicinally by the native practitioners of the Indian peninsula from time immemorial. The seeds are of a convex shape on one side, and smooth and angular on the other. They are reckoned by the natives amongst their drastic purges, and are frequently prescribed by them in maniacal cases, or on other occasions when powerful purgatives are indicated. Their operation is rendered much less violent when the seeds are cleansed from the thin filament in which each is closely enveloped: then as much as one of the seeds may be given as a dose. A fixed oil is extracted from the seeds by the natives, called *Nervalum unnay.* It is considered a very valuable external application in rheumatic affections; but, when used in this way, it is generally mixed with some bland oil, to counteract its acrid effect; and, as far as I could learn, the oil has never been administered internally by the natives.

The way to prepare the seeds is as follows:—Take the seeds of the croton *(grana tiglia)*, which, after being each enveloped by a small ball of fresh buffaloe's dung, about the size of a sparrow's egg, put upon some burning charcoal till the dung is burnt dry; then removing them, and taking off the shells from the seeds, pound the nuclei, and divide into two pills,—viz. two out of each grain of the mass. The mass is generally mixed with some honey, to give it bulk and consistency. The advantage derived from the abovementioned process is that, in the first place, it facilitates the removal of the shells; secondly, it renders the nucleus more fit for pounding; and, thirdly, the gentle torrefaction it undergoes corrects, in a great degree, its natural acrimony. One pill of the above is sure to operate; two pills a full dose: an excess in the dose acts violently on the stomach. It is a powerful evacuant of the bile; and, by the Malays, is administered successfully as a hydragogue.

The root of this was formerly regarded, at Batavia and Amboyna, as a specific in dropsy, taken infused in wine.

At Benares, they boil the seeds soft in milk, stripping them first of the shells; after which they pound them, forming the mass by means of lime-juice, at the rate of one pill from each seed: two of these pills are considered a full dose. A mode in Guzerat is still more simple, and consists merely in pounding the kernels, without any previous operation, and forming, by means of honey, from each nucleus two pills; one of which generally suffices for a drastic purge, directing a gill of warm water to be taken immediately after swallowing the pill. In this preparation, the inherent acrimony of the kernels makes up for the smallness of the dose; and the water drank upon it insures, they say, its speedy operation downwards.

The chief advantage of these pills appears to me to be the smallness of the bulk necessary to obtain the desired effect. None of the drastic purges are so certain; none more rapid in their action; and none, I think, so little annoying by griping or nausea. Five hundred doses may easily be contained in an ordinary pill-box.

The expressed oil of the seeds has been of late introduced into practice in Europe, in cases where drastic purges are indicated. Dr. Conwell, of the Honourable Company's Madras establishment, was the first who sent it to England; at the same time he contrived to have a small quantity conveyed to the principal universities on the continent of Europe, with the view of bringing this powerful medicine into notice. A considerable quantity of the oil has been lately imported, per ship *Hindostan*, from Madras, and consigned to Mr. Short, apothecary, in Ratcliffe Highway. One-third to one-half a drop of the oil, if good, is a full dose.

*Madras; 12th May,* 1822.

